# The Past, Present and Future of Flow Cytometry in Central Nervous System Malignancies

**DOI:** 10.3390/mps4010011

**Published:** 2021-01-26

**Authors:** Evrysthenis Vartholomatos, George Vartholomatos, George A. Alexiou, Georgios S. Markopoulos

**Affiliations:** 1 Faculty of Medicine, Neurosurgical Institute, School of Health Sciences, University of Ioannina, 45110 Ioannina, Greece; eyrys.varth@gmail.com (E.V.); galexiou@uoi.gr (G.A.A.); 2 Haematology Laboratory-Unit of Molecular Biology, University Hospital of Ioannina, 45110 Ioannina, Greece; gvarthol@gmail.com; 3 Department of Neurosurgery, University of Ioannina, 45110 Ioannina, Greece

**Keywords:** central nervous system malignancies, flow cytometry, glioblastoma, intraoperative flow cytometry, phenotypic analysis, DNA content analysis

## Abstract

Central nervous system malignancies (CNSMs) are categorized among the most aggressive and deadly types of cancer. The low median survival in patients with CNSMs is partly explained by the objective difficulties of brain surgeries as well as by the acquired chemoresistance of CNSM cells. Flow Cytometry is an analytical technique with the ability to quantify cell phenotype and to categorize cell populations on the basis of their characteristics. In the current review, we summarize the Flow Cytometry methodologies that have been used to study different phenotypic aspects of CNSMs. These include DNA content analysis for the determination of malignancy status and phenotypic characterization, as well as the methodologies used during the development of novel therapeutic agents. We conclude with the historical and current utility of Flow Cytometry in the field, and we propose how we can exploit current and possible future methodologies in the battle against this dreadful type of malignancy.

## 1. Introduction

Carcinogenesis is the step-by-step process through which normal cells acquire genetic and epigenetic alterations and transform into malignant cells that form a tumor mass. Cancer is among the leading causes of human mortality worldwide, with 18.1 million new cases and 9.5 million deaths in 2018 [1]. Among them, central nervous system malignancies (CNSMs), including brain tumors (ICD codes C70-72) account for ~308,000 new cases and ~251,000 deaths, making it one of the deadliest types of cancer per case [1]. Central nervous system tumors have been historically classified on the basis of the histology parameters, mainly as a result of the occurrence of malignancy from different brain tissues [2]. However, the latest classification of CNSMs, the 2016 update from the World Health Organization, takes into account more advanced molecular characteristics, that are now available in the post-genomic era, providing a more comprehensive catalog with usefulness in clinical management and treatment [3].

Glioblastoma (GBM) is the most common malignant primary brain tumor. Despite intensive clinical investigation and several novel therapeutic approaches, the patient’s median survival remains poor, in the range of 15 months [4]. The standard treatment approach involves surgical resection followed by radiotherapy with concurrent and adjuvant chemotherapy [5]. Many chemotherapeutic agents have been used against GBM including, among others, temozolomide (TMZ) [6]. However, the genetic heterogeneity and the diverse molecular pathology make it difficult to successfully treat the GBMs, and cells that are not eradicated eventually grow, and virtually all recurring tumors are resistant to both chemotherapy and radiotherapy [7].

Treatment of CNSMs pose many challenges. First, the central role of the brain in body homeostasis is a major obstacle for surgical removal, since the removal of healthy tissue may result in serious side effects. In other words, a brain surgery should be performed with removal of the tumor, without affecting the adjacent normal tissue. Second, the same is true for radiotherapy. Third, commonly used chemotherapeutics often do not pass the blood-brain-barrier, which renders them inefficient for treatment of CNSMs.

Flow Cytometry (FC) is a powerful analytical technique with several applications in phenotypic analysis and the quantification of cellular processes, such as proliferation and cell death [8]. The quantification of state/phenotype of a cell population is among the main advantages of FC over other methods, such as microscopy. Among the main limitations of the methodology is the spectral overlap of fluorochromes and the inability to detect the intracellular localization of labeled targets. New advances in the field, such as the development of mass cytometry [9,10] and spectral Flow Cytometry [11] allow the simultaneous analysis of several parameters in a single cell, overcoming spectral overlap of traditional cytometry. In addition, Imaging Flow Cytometry [12,13] and more recently imaging mass cytometry [14] combine the analytical potential of cytometry with the imaging abilities of a microscope.

**Figure 1:**
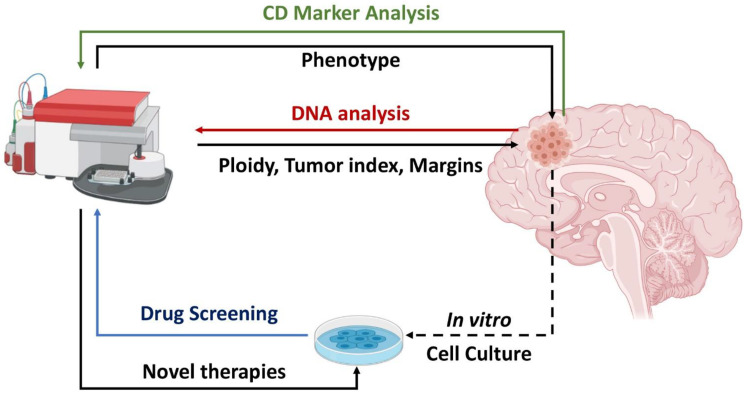
Utility of Flow Cytometry in central nervous system malignancy (CNSM) analysis. CNSM cells can be directly analyzed intraoperatively or post-surgical for DNA content (red arrow), offering information on ploidy, tumor index, and tumor margins. CNSM analysis for Cluster of Differentiation (CD) markers (green arrow) can provide information on tumor phenotype and possibly on the clinical outcome. Analysis of in vitro CNSM cultures (blue arrow) may offer insights on drug efficacy. Created with BioRender.com.

In the following sections we discuss the utility of Flow Cytometry in diagnosis and treatment of CNSMs. In each section, a summary of past and present methods is presented, followed by a perspective of possible future applications. The concepts of the study are summarized in Figure 1.

## 2. DNA Content Analysis by Flow Cytometry in Brain Malignancies

### 2.1. History and Early Analysis

DNA analysis is among the first widely used applications of FC, even in the era before the development of methodologies for monoclonal antibodies [15]. Early studies in the field of FC and DNA-content analysis, in particular, demonstrated the ability to quickly characterize cell populations of human tumors with FC and suggested the possible usefulness for clinical management of cancer.

Importantly, cell cycle phase distribution and cell ploidy by DNA content analysis of CNSMs has been established more than 40 years ago. Frederiksen et al. have established a protocol to study DNA content distribution in a number of 85 patients with either benign or malignant brain tumors. Diploid DNA content was observed mostly in inflammatory lesions and most of the benign tumors. Malignant tumors had been characterized to contain hyperploid DNA content. Interestingly, this study outlined the importance of Flow Cytometry as a fast and robust method for analysis of CNSMs [16]. Kawamoto et al. have used low cytometric analysis for DNA content in cells of normal human brain and in benign and malignant CNSMs. They found that loss-of-heterozygosity is analogous to the CNSM stage. On the basis of a small number of samples, they proved the validity and were among the first to propose the utility of Flow Cytometry and DNA distribution as data with clinical importance in cancer in general and in CNSMs diagnosis in particular [17]. Hoshino et al. provided similar data on several types of CNSMs. The main methodological differences of this and the aforementioned method is the purification of nuclei taken from CNSMs centrifugation through 40% sucrose and staining with an acriflavin-Feulgen reagent for DNA analysis. The results were similar, since the DNA content in the benign tumors (meningiomas, pituitary adenomas, neuroblastomas, and low-grade astrocytomas) showed mainly diploid cell populations with a low proliferation index, while most the malignant CNSMs, which were mainly gliomas, had aneuploid populations and/or a higher proliferation index [18]. Another interesting insight has been added by the study by Petersen et al., in which a rare CNSM has been characterized by both Flow Cytometry and cytogenetic analysis as hypodiploid, containing about 75% of the normal amount of DNA of a diploid cell [19]. The use of Flow Cytometry has also been proved useful to not only solid brain tissue but also for the analysis of cerebrospinal fluid when there is infiltration by pathological cells [20].

### 2.2. Development of DNA Content Analysis

For several years, research in the field has been fruitful and confirmed the first observations, and further developments, such as the analysis of proliferative potential, has provided novel data. A good paradigm of such developments is the work from Nishizaki et al. [21]. This research team has assessed proliferation potential by quantifying a monoclonal antibody for the proliferation marker Ki-67, the labeling of 5-bromodeoxyuridine (BRdU), a thymine analog, that is incorporated to newly synthesized DNA during DNA replication in the S-phase. These two markers were used along with DNA staining in 48 human CNSMs. Notably, both Ki-67 and BRdU were correlated with the degree of malignancy, which was based on conventional histological analysis. DNA content analysis was also associated with both labeling indices, indicating a possible clinical importance [21]. From another point-of-view, Danova et al. have assessed the possible use of propidium iodine (PI) and BRdU to label cells in vivo, by infusing non-toxic concentrations on BRdU in patients. Following the analysis of 22 clinical cases with both benign and malignant CNSMs, they observed no immediate toxicity from BRdU administration. There has been no follow-up study, hence the possible long-term health effects of BRdU have not been assessed by the authors. BRdU incorporation was significantly different between benign and malignant CNSMs, with glioblastomas containing at least 2–3 times larger fractions of their cell populations in the S-phase of the cell cycle. The authors’ data suggested that, in vivo, use of BRdU may allow the use of FC in clinical settings, in the assessment of prognostic significance of different proliferative parameters during CNSM characterization and treatment [22]. Following the same conceptual approach, Crone et al. have found that Flow Cytometry is useful as a diagnostic and also as a prognostic tool in human meningiomas. In the same study, the malignancy has been associated with proliferative potential and aneuploidy as well as with cerebral edema [23].

During the decade of 1990–2000, there have been reports that confirmed the utility of DNA content analysis in CNSMs. Report of a rare case analysis of a ganglioma included Flow Cytometric and cytogenetics analysis. Flow Cytometric analysis of DNA content revealed a higher mitotic index compared to normal tissue. Tumor cytogenetics on short term cultures confirmed a complex abnormal karyotype with several structural chromosomal abnormalities [24]. Several other studies proved that DNA content is diagnostic for several CNSMs [25], such as as well as prognostic marker for oligodendroglioma [26] and possibly for choroid plexus tumors [27]. Remarkably, two studies found that DNA content analysis may be also suitable for the study of intratumoral heterogeneity and the differences in therapy resistance [28], as well as regional heterogeneity on DNA content [29] in gliomas. The results of both studies are in agreement with the concept of clonal evolution in cancer, in which the Darwinian evolution of a benign lesion to a malignant and metastatic cancer leads to the development of several clones, each one of them containing different characteristics and traits in a “struggle for existence” [30].

### 2.3. Intraoperative Flow Cytometry

In spite of the potential of clinical utility, Flow Cytometric analysis of DNA content and proliferation markers in CNSMs diagnosis have been scarcely used in clinical practice either as a diagnostic or a prognostic tool. This was until the development of a novel concept, which is the intraoperative use of Flow Cytometry for DNA content/ploidy and cell cycle distribution analysis. The development of Intraoperative Flow Cytometry (iFC), during the last decade, offered a novel viewpoint and perspective on the utility of DNA content analysis for the characterization of solid tumors which have not been extensively evaluated, such as hematologic malignancies. The rationale of iFC offered the ability for intraoperative diagnosis, as an alternative to the pathology evaluation of tissue sections obtained during surgery. A modified rapid protocol for cell cycle analysis developed in the University Hospital of Ioannina (Ioannina Protocol) allowed the intraoperative characterization of intracranial lesions and their surgical margins in 6 min per sample. In a study with thirty-one patients, a significant difference in the G0/G1 phase, as well as in S-phase and G2/M fractions between high-grade and low-grade tumors, was demonstrated. In glioblastoma patients, significant differences were found between tumor mass and margins regarding the G0/G1 phase, the S-phase, and (G2/M) tumor fraction (Tumor Index), offering the potential of delineating tumor margins in gliomas [31]. The Ioannina protocol was first established in a retrospective study involving a series of tumor samples taken from 56 patients, during surgery. The results of DNA content analysis showed that the cell cycle distribution analysis could differentiate between grade I from grade II/III meningiomas and low from high grade gliomas. Furthermore, a prognostic significance was found in glioma patients, based on the analysis of clinical results over a 5-year period [32]. Intraoperative cell-cycle analysis of CNSMs, based on the Ioannina protocol has been suggested as an alternative to other novel intraoperative diagnostic techniques, such as mass-spectrometric analysis of tumor metabolites [33,34], the use of 5-Aminolevulinic Acid Fluorescence (5′ALA), and Intraoperative Magnetic Resonance Imaging [35,36,37], as well as intraoperative squash smear cytology [38,39]. Among the advantages are time, sensitivity, and specificity.

A research team from Tokyo Women’s Medical University has independently developed a similar, rapid iFC protocol with an analysis time of 10 min per sample. The results from Shioyama et al., using their iFC protocol in 328 biopsy specimens of gliomas, revealed an optimal mitotic index of 6.8%, resulting in 88% sensitivity, 88% specificity, 97% positive predictive value, 60% negative predictive value, and 88% diagnostic accuracy [40].

A joint publication by members of both groups highlighted that iFC is a promising adjunct for intracranial tumor surgery, may aid the identification of gliomas boundaries, it can identify a tumor’s grade, diagnose lymphoma, and has prognostic value in glioma [41]. As regards prognosis, recent data suggest that the calculation of of the malignancy index, based on iFC, may also act as a novel prognostic factor following radiotherapy and chemotherapy with temozolomide [42].

We believe that in the future, the utility of DNA content analysis is going to be expanded into three different directions, based on emerging trends, during the last years. First, the vision of real-time flow-cytometry analysis is on the verge of becoming a reality [43,44], diminishing the analysis time into seconds (from currently 6 min per sample). Second, the utility of iFC is currently being expanded beyond CNSMs into the analysis of tumor margins in several additional cancer types [45], with candidates such as head-and-neck malignancies [46,47] and breast cancer [48]. Third, as described in the following sections, DNA content analysis may be combined with several other parameters that can be quantified by Flow Cytometry. A good paradigm of candidate multiparameter-flow-cytometry analyses is the detection of brain lymphomas, since they can be quantified by intraoperative DNA content analysis [49] and also by immunophenotypic characterization [50]. Another good example is the use of the CD56 marker, which has been proved useful for pediatric CNSMs grading [51], along with DNA content analysis, for a more accurate diagnosis [52].

## 3. Phenotypic Analysis

Flow Cytometry represents the gold standard methodology for quantitative, cell-specific phenotypic analysis [8]. The development of cytometry has enabled the diagnostic analysis of hematological malignancies, guiding therapy and follow-up of a patient for the possibility of Minimal/Measurable Residual Disease [53,54,55,56].

Cluster of Differentiation (CD) antigens have been extensively used in cancer research. CD antigens represent surface markers corresponding to proteins with crucial roles in cell-cell adhesion and interaction, signaling, and differentiation [57]. A good paradigm is the expression of markers CD44 and CD24 in cancer. CD44 is a cell-surface glycoprotein involved in cell–cell interactions, cell adhesion, and migration and has long been referred to as HCAM (homing cell adhesion molecule). CD24 is a signal transducer CD24, also known as heat stable antigen HSA. Initially, breast cancer cells with a CD44+/CD24- phenotype were characterized as cancer stem cells (CSCs) [58]. The two markers have been found to be differentially expressed in different types of breast cancer and their expression has also been associated with distinct clinical outcomes, providing a possible prognostic significance [59]. The same is true for several other markers including CD133 (also known as prominin-1, a member of pentaspan transmembrane glycoproteins, which specifically localize to cellular protrusions) [60], CD90 (or Thy-1, glycophosphatidylinositol (GPI) anchored conserved cell surface protein, a marker for a variety of stem cells and for the axonal processes of mature neurons), and CD34 (first described on hematopoietic stem cells as a cell surface glycoprotein that functions as a cell-cell adhesion factor) [61].

There have been several reports regarding the phenotypic characterization of CNSMs. CD133 has been originally identified as a marker of early hematopoietic stem cells as well as neural stem cells [62]. CD133 is also among the most established phenotypic markers of cancer stem cells in human brain tumors and among the first markers to be associated with any type of cancer [63]. The expression of CD133 is associated with self-renewal proliferation, as well as differentiation capacity. CD133, along with Sox2, musashi-1, and bmi-1, and phosphoserine phosphatase has also been associated with CSCs in pediatric CNSMs [64]. Interestingly, CNSMs may contain CD133 positive or negative cells which exhibit differential growth characteristics [60], different transcriptional profiles which suggest different cells of origin [65], and implicate different strategies for personalized therapies [66]. Since cancer stem cells are critical for cancer development, therapeutic strategies take into account the depletion of CD133+ cells in CNSMs. In one such effort, Notch pathway has been found more active in CD133+ CNSMs and notch pathway blockade by γ-secretase inhibitors reduced neurosphere growth both in vitro and in ex vivo xenografts [67].

CD15, also known as stage specific embryonic antigen-1 (SSEA-1), is known to play roles in cell-to-cell recognition processes and has been originally associated with Hodgkin and non-Hodgkin lymphoma and some cases of leukemia [68]. An early report showed that CD15 is expressed in CNSMs [69]. CD15 has been later confirmed as a hallmark of tumor-initiating cells in human glioblastoma [70]. The role of CD15 in early neurogenesis has been also elucidated, since the expression of CD15 is part of a neural lineage-differentition specific code that also includes CD24 and CD44 [71]. A Flow Cytometry assay has been developed to identify neural cell tissue, by analyzing the co-expression of CD133, CD15 and CD24 [72].

CD56 is a neural cell adhesion molecule (NCAM), that has been initially found to be expressed in normal neural cells, as well as in CNSMs [73]. A recent report quantified CD56 expression in tissues from 46 pediatric brain tumor cases, using the methodology of Flow Cytometry. A significant negative correlation between Ki-67 index and CD56 molecules/cells was exhibited. Additionally, CD56 was diagnostic of CNSMs, since normal brain tissue could be differentiated from CNSMs on the basis of CD56 expression, while there was also grade specific differences in CD56 expression [51]. In another study, cell cycle analysis by propidium iodine was used in combination with staining of CD56+ cells by Flow Cytometry. This method could accurately distinguish CNSMs and non-neoplastic tissue, as well as high-grade from low-grade CNSMs. Half of the CNSMs had a non-diploid DNA profile, while all CNSMs exhibited significantly lower G0/G1 than normal brain tissue. Additionally, low-grade tumors had a significant lower S-phase than high grade tumors. Grade IV tumors had the lowest G0/G1 fraction, and this was adequate to be distinguished from grade III tumors. Flow Cytometric analysis of cell cycle distribution in CD56+ cells has been able to determine malignancy and has been proposed as a possible novel adjunct diagnostic technique to histopathological evaluation in pediatric brain tumors [52].

Mass Cytometry (MC) is a recent advance in the field of Cytometry, that has been able to simultaneously analyze up to 36 markers in several cancer types, including glioma. Among them, CD45, CD3, CD4, CD8, CD19, CD64, HLA-DR, CD11c, CD56, CD44, GFAP, S100B, SOX2, nestin, vimentin, cytokeratin, and CD31 have been differentially expressed in different cancer subtypes [74]. An advantage of the method is the distinction of cancer cells to cells of tumor microenvironment, providing an accurate depiction of the factors affecting cancer promotion. In a publication by Vasquez et al., immunity to SOX2 in glioma patients has been assessed utilizing a single-cell MC-based 37-parameter panel [75]. The deep profiling capabilities of mass cytometry is also elucidated in the potential to profile the phenotype of brain tumor initiating cells in the context of GBM [76], while the combination of MC with RNA-seq has allowed the mapping of cellular states of microglia, providing valuable information in the context of how gliomagenesis may occur [77]. Recent applications of MC have contributed towards the role of the microenviroment in CNSM homeostasis as well as towards the development of immunosuppressive phenotypes, which are distinct depending on the tumor type [78,79,80,81,82].

## 4. Flow Cytometry for Study of Anticancer Agent Efficacy

CNSMs constitute life-threatening neoplasms, based both on the aggressive phenotype as well as the nature of normal brain tissue. The surgical removal of CNSMs comes with many difficulties that have been addressed in the previous section. Following surgery, or in some cases where surgical removal is not an option, the two main approaches are radiotherapy and chemotherapy.

The chemotherapeutic agents that are currently used in clinical practice are limited compared to other tumor types. The blood brain barrier that prevents the entry of high molecular weight substances is a major factor posing problems to treatment. Hence, the development of novel small molecule therapeutics to overcome the blood brain barrier may be an approach to a better treatment of CNSMs. Although sometimes morphologically similar, CNSMs have different clinical outcomes, which can be partially explained by different tumor molecular fingerprints. The heterogeneity of CNSMs, which is more prominent in GBMs, is among the main challenges underlying therapeutic failure, as GBMs undergoing conventional treatment regimens eventually become resistant [3,83]. Genetic profiling appears to separate GBMs which arise de novo (primary GBMs) from those arising from pre-existing low-grade diffuse gliomas (secondary GBMs). Primary and secondary GBMs show similar histological characteristics but they differ in genetic and epigenetic profile [84,85,86]. The biological distinction of CNSM subgroups is crucial for guiding the design of clinical trials [86]. GBM is a heterogeneous brain tumor with evident pathological and genomic variants [87,88]. The MGMT (O6-methylguanine-DNA methyltransferase) protein, encoded by the *MGMT* gene, plays an important role in repairing the DNA damage from alkylating chemotherapeutic agents (e.g., temozolomide). MGMT promoter methylation has both prognostic and predictive significance, since it has been associated with longer survival rates in patients treated with chemo-radiation and subsequent adjuvant temozolomide [89]. Glioblastoma cells may exist in several different cellular states, with characteristics of either neural progenitors cells (NPC), oligodendrocytes, astrocytes or mesenchymal cells (MSC) [90], that are associated with different genetic alterations, each favoring a particular cellular state. The potential for a single cell to generate all four states has been also associated with cancer stem cell (CSC) markers CD24 and CD44, which are among the four top-scoring genes for the NPC-like and MSC-like states, respectively [90]. Orally administered TMZ, is the first-line treatment for GBM, since it prolongs survival and delays progression without impacting on the quality of life. Even in the elderly, TMZ is comparable to radiotherapy regarding overall survival and progression-free survival [6]. Since TMZ acts as an alkylating agent, GBM acquires resistance to TMZ, which is controlled by the expression of DNA repair protein MGMT, which is a major obstacle to GBM treatment [91,92]. A suitable in vitro cellular model on the effects of TMZ on GBM and its mechanisms of resistance are the GBM cell lines U251MG and T98G. The former does not express MGMT protein and it is TMZ- sensitive, whereas the latter express significant amounts of MGMT and are resistant to TMZ [93].

Flow Cytometry has been proved to be a valuable tool in both phenotypic characterization and the efficacy of various chemotherapeutic agents (Figure 1). Using U251MG and T98G cells, in a previous report we uncovered an additional mechanism of TMZ action. TMZ was shown to alter the expression of several CSC markers, including CD15, CD133, CD24, CD44, and nestin [94]. TMZ resulted in the increased expression of CD15, a marker which is downregulated in low grade gliomas [69], while it also altered the expression of CD24, CD44, and nestin [95].

While there has been extensive research on novel therapeutics of GBM, these treatments have not entered clinical trials, and their impact was exploited only in cellular glioblastoma models. Our research group has been working towards novel therapeutics of GBM, which are based on natural products. Specifically, on the basis of experimental results that include FC methodologies, we have found that difluoromethylornithine (DFMO) [96] as well as the natural substances moschamine [97], n-p-coumaroyl-serotonin [98], and deglucohellebrin [99] exhibit significant antiglioma activity in vitro and low cytotoxity in vivo, as shown in a zebrafish embryo model.

The contribution of Flow Cytometry in the field is substantial, since it provides a toolbox of cell-specific assays. DNA content and cell cycle analysis provide data on cell cytotoxicity, as a means to prove the efficiency of a chemotherapeutic agent. The dual stain of annexin/PI is the gold-standard for apoptosis quantification, while the different agents may act with different mechanisms. DNA content analysis revealed several mechanisms of action for different antiglioma agents. For example, N-(p-coumaroyl)-serotonin resulted in both S and G2/M phase arrest, moschamine resulted mostly in S phase arrest and deglucohellebrin in G2/M phase arrest [97,98,99]. Several other studies have used in DNA content analysis quantification in several CNSM treatments, revealing, among others, the antiglioma effects of curcumin [100], quercetin [101], glycyrrhizic acid [102], and palbociclib [103]. Recently, the antiglioma effect of drugs of the class of antipsychotics have been tested using, among other assays, Flow Cytometry techniques. It has been revealed that the repurposing of antipsychotic drugs, such as haloperidol [104] and phenothiazine [105], may be a viable alternative for the treatment of CNSMs. Another recent report utilizing Mass Cytometry, revealed the selective targeting of glioma stem cells (characterized as CD98^+^ cells coexpressing stem cell markers, including Oct3/4, Nestin, SOX2, Musashi-1, PDGFRα, Notch2, Nanog, STAT3 and C-myc) by P-boronophenylalanine (BPA), a chemical compound used in Boron neutron capture therapy [106].

Phenotypic analyses revealed the effects of different treatments on cell behavior and possibly a mechanistic approach to chemoresistance. Following treatment of GBMs with conventional therapies, the use of multi-color Flow Cytometry revealed a chemo-resistant population that is CD44 positive, which was also correlated with poor outcomes of the disease [107]. Multicolor Flow Cytometry analysis of 9-marker multicolor panels in GBM patients (CD133, CD44, CD15, A2B5, CD36, CXCR4, IL6R, L1CAM, and ITGA6) revealed a phenotypic signature of CD44+/CD133+/ITGA6+/CD36+ of CSCs. Patients with an enriched population of the above signature had a significantly worse survival outcome, revealing a possible novel pathway for targeted therapeutics [108]. In another study, multicolor Flow Cytometry revealed that CD8+ and CD38- immune effector cells in the site of cancer are associated with better survival, following treatment [109].

## 5. Conclusions

Flow Cytometry is the gold standard approach for quantitative phenotypic analysis at the cellular level. The utility of Flow Cytometry in CNSMs is multilayered. DNA content analysis has been used from the bench to the operating theater and is evolving as a novel diagnostic approach to assess malignancy and to validate excision margins. Phenotypic analyses by Flow Cytometry have been able to delineate complex phenotypes during carcinogenesis and cancer stem cell formation. Following treatment, cytometry has been used both in in vitro and in a clinical setting to evaluate the efficiency of current and novel therapeutics. The developments in the field are in parallel with the development of novel therapeutic and diagnostic approaches, and we believe that in the future, these will further improve survival in patients with CNSMs.
